# Early modulation of autophagy-associated markers in murine adenomyosis and selective persistence in human stromal lesions

**DOI:** 10.3389/fendo.2026.1750026

**Published:** 2026-03-25

**Authors:** Julie Vervier, Marlyne Squatrito, Silvia Blacher, Michelle Nisolle, Carine Munaut

**Affiliations:** 1 Laboratory of Tumor and Development Biology, Giga-Cancer, University of Liège, Liege, Belgium; 2 Obstetrics and Gynecology Department, University of Liège, Liege, Belgium

**Keywords:** adenomyosis, autophagy-associated markers, endometrial stroma, endometrial–myometrial interface, immunohistochemistry, LC3B, tamoxifen-induced murine model, uterine pathophysiology

## Abstract

**Background:**

Adenomyosis is a heterogeneous uterine disorder characterized by the presence of endometrial tissue within the myometrium. Autophagy, a key homeostatic process involved in tissue remodeling and stress adaptation, has been inconsistently described in adenomyosis. This study aimed to define the temporal and compartment-specific regulation of autophagy-associated markers during disease progression, using a tamoxifen-induced CD1 mouse model and human uterine samples.

**Methods:**

Female neonatal CD1 mice were exposed to oral tamoxifen from postnatal days 1–4 and evaluated at 1 and 3 months. Uterine tissues were examined for autophagy- and apoptosis-related transcripts (RT-qPCR), autophagy-associated protein expression (Western blot) and LC3B distribution (immunohistochemistry and immunofluorescence). LC3B expression was also assessed in paraffin-embedded human uterine samples from women with histologically confirmed adenomyosis and from controls without uterine pathology.

**Results:**

At one month, a restricted set of autophagy/apoptosis-related transcripts (*Akt1, Mapk1, Nfkb1, Cxcr4, Bax, Bcl2, Bcl2l1 and Ulk2*) was modulated, and LC3B protein levels were decreased, coinciding with early myometrial disorganization and stromal invasion. By three months, gene and LC3B staining differences were no longer detectable by the assays used, despite established lesions, suggesting biological adaptation or involvement of compensatory pathways. In human adenomyosis tissues, LC3B reduction was confined to the stromal compartment of ectopic lesions, whereas glandular and eutopic endometrium remained comparable to controls, indicating a persistent stromal-specific alteration of autophagy-associated markers.

**Conclusions:**

Modulation of autophagy-associated markers is an early and transient event in murine adenomyosis initiation, whereas in human disease, stromal-specific LC3B reduction persists in established lesions. These findings indicate that autophagy-associated regulation is temporally and spatially distinct in adenomyosis and suggest that stromal autophagy-associated dysregulation may represent a pathway for future therapeutic exploration, while further studies are required to determine its functional and causal relevance.

## 1 Introduction

Adenomyosis is a chronic benign condition defined by the ectopic presence of endometrial glands and stroma within the uterine myometrium, usually surrounded by reactive smooth-muscle hyperplasia (1). The disease affects approximatively one in five women of reproductive age and is associated with abnormal uterine bleeding, pelvic pain and infertility (2). These symptoms, however, lack specificity and frequently overlap with those of endometriosis and uterine myomas which often coexist with adenomyosis and confound both diagnosis and mechanistic inference (1).

Multiple hypotheses have been proposed to explain lesion initiation, including invagination of the endometrial basalis into the myometrium though mechanisms related to tissue-injury and repair, metaplasia transformation of Müllerian remnants, aberrant differentiation of displaced endometrial/stromal stem cells, enhanced invasion across the endometrial–myometrial interface (EMI), local hormonal dysregulation, and genetic/epigenetic alterations such as KRAS mutations (3–5). Disease progression may further involve the epithelial-mesenchymal transition (EMT), inflammation, hormonal imbalance, altered PI3K/AKT/mTOR and autophagic-associated regulation (4, 6). Data on the role of autophagy in adenomyosis remain scarce and elucidating these mechanisms in patients is hindered by phenotypic heterogeneity and frequent comorbidities.

Controlled animal models provide a tractable framework to dissect early disease mechanisms under defined hormonal and experimental conditions. We therefore used a tamoxifen-induced CD1 mouse model to characterize the temporal regulation of autophagy during disease development. While CD1 mice can spontaneously develop adenomyosis after 6 months at low incidence, neonatal tamoxifen exposure dramatically increases penetrance, improving reproducibility and the confounding effects of coexisting uterine pathologies (7).

Macroautophagy (hereafter referred to as “autophagy”) is a conserved cellular recycling process in which LC3-decorated autophagosomes sequester cytoplasmic cargo for lysosomal degradation (8). Autophagy maintains cellular homeostasis, adapts metabolism to stress, and intersects with development, immunity, inflammation, and cell death (9). While primarily cytoprotective, excessive self-degradation can be harmful, and defective autophagy has been implicated in diverse human pathologies, including neurodegenerative and metabolic diseases, cancers, ageing, inflammatory and autoimmune disorders (10). In the endometrium, autophagy is hormonally regulated: estrogen generally restrains it, whereas progesterone promotes autophagic activity during the secretory phase. Autophagic activity peaks late in the menstrual cycle, contributing to controlled apoptosis and shedding (11), and supports endometrial receptivity and implantation (12). Dysregulated autophagy has been reported in endometrial hyperplasia, carcinoma, infertility, and endometriosis (12). In adenomyosis, however, available studies show heterogeneous and sometimes contradictory findings, and whether autophagy contributes lesion initiation or maintenance remains unclear (13).

The aim of this study was to investigate the contribution of autophagy to the development of adenomyosis across experimental and clinical settings. Using a neonatal tamoxifen-induced CD1 mouse model, we evaluated autophagy at an early pre-lesional stage (1 month) and at a lesion-established stage (3 months), thereby capturing different phases of disease evolution. To bridge these findings to the human condition, we analyzed uterine specimens from women with histologically confirmed adenomyosis obtained at hysterectomy, representing advanced disease.

## 2 Materials and methods

### 2.1 Mouse model of adenomyosis and estrus cycle stage assessment

Animal experiments were approved by the Animal Ethics Committee of the University of Liège (#2387) and conducted in strict accordance with institutional guidelines for the care and use of laboratory animals. Mice were kept under controlled conditions of temperature (+/- 21 °C) and light (12h/12h light/dark cycle) with ad libitum access to food and water. Animal welfare was monitored daily.

Female neonatal CD1 mice, obtained from pregnant dams provided by Charles River Laboratories (Italy), received oral doses of 2.7µmol/kg tamoxifen (=1mg/kg) (Sigma, T5648) suspended in a mixture of peanut oil, lecithin, and condensed milk (2:0.2:3, v/v) from postnatal days 1 to 4 after birth (day of birth = day 0). Control mice received vehicle only. As previously reported by our group and others, this protocol induces adenomyosis with a high penetrance (> 98%) (14).

To minimize hormonal variability affecting on autophagy, only mice in the proliferative stage of the estrus cycle (proestrus or estrus phase) were analyzed. Estrous cycle stage was used as an inclusion criterion and not as an experimental outcome. Cycle synchronization was achieved by 3 consecutive daily subcutaneous injections of 17ß-estradiol (100ng/100µl) prior to sacrifice (15). On the day of sacrifice, estrous stage was confirmed by vaginal cytology according to *Caligioni* (16). Briefly, vaginal lavage samples were smeared on glass slides, dried, fixed in 100% ethanol (30 min), rehydrated, and stained with hematoxylin–eosin. The stage was determined by the relative abundance and morphology of leukocytes and epithelial cells under light microscopy (40×), as described by Byers et al. (17).

### 2.2 Tissue collection

The mice were euthanized at either 1 or 3 months old, and uteri were collected. In 1-month-old mice, one uterine horn was divided into 2 parts, while in 3-month-old mice it was divided in 3 parts, which were snap-frozen for RNA and protein extraction. The contralateral horn was fixed in 4% formalin and paraffin-embedded. Histological analyses were performed on hematoxylin and eosin (H&E) stained sections, as well as by immunofluorescence (IF) and immunohistochemistry (IHC), as described below.

Human paraffin-embedded uterine samples were obtained by the Biothèque Hospitalo-Universitaire de Liège (BHUL) from anonymized hysterectomy specimens. Adenomyosis cases (mean age 43.2 years) were selected based on histological confirmation. Control samples (mean age 29 years) were derived from hysterectomies performed for gender-affirming surgery or prophylactic hysterectomy, with no underlying uterine pathology. The study was conducted under institutional and national ethical approval (CE1267).

### 2.3 Real time PCR analysis for mRNA

Total RNA was extracted from individual uterine samples using the RNeasy Mini Kit for tissues (Qiagen) following the manufacturer’s instructions. RNA concentration and integrity were assessed and 1µg samples was reverse transcribed into complementary DNA (cDNA) using FastGene Scriptase II Ready mix (Nippon Genetics) according to the manufacturer’s instructions. Primers were designed and purchased from Eurogentec (**Table 1**). RT-qPCR was performed using SYBR-green PCR Master Mix on an Applied Biosystems 7500 Fast Real-Time PCR System (QuantStudio 3, Thermo Fisher Scientific).

**Table 1:** Primers for qRT-PCR.

Gene name	Primer sequences (5’ – 3’)	Product size (bp)
Atg9b	Forward: GAACCATACAAGACGTGGACCAT Reverse: CCCAGGGTACCGAGCTGAG	248
Akt1	Forward: TCGTGTGGCAGGATGTGTAT Reverse: ACCTGGTGTCAGTCTCAGAGG	78
Bcl2	Forward: AACATCGCCCTGTGGATGAC Reverse: TATGCACCCAGAGTGATGCAG	191
Bcl2l1	Forward: GCCACCTATCTGAATGACCACC Reverse: AGGAACCAGCGGTTGAAGCGC	134
Bid	Forward: GCGTCTGCGTGGTGATTC Reverse: TCCCAGTAAGCTTGCACAGG	211
Cxcr4	Forward: CCATGGAACCGATCAGTCTGA Reverse: CGGTACTTGTCCGTCATGCT	238
Dapk1	Forward: GGCGCACGAAGCGAC Reverse: AGACCGGTACTTTTCTCACGAC	245
Igf1	Forward: AAGCGATGGGGAAAATCAGC Reverse: ATAGCCTGTGGGCTTGTTGAA	242
Lc3b	Forward: GTCCTGGACAAGACCAAGTTCC Reverse: CCATTCACCAGGAGGAAGAAGG	119
Pik3c3	Forward: TGCAGTTCATCCAGTCGGTT Reverse: TCACACAGTATCCAGCACAGC	159
Tnfs10	Forward: GGAAGACCTCAGAAAGTGGCAG Reverse: TTTCCGAGAGGACTCCCAGGAT	129
Ulk1	Forward: GCATCGAGCAAAACCTGCAA Reverse: CCACTTGGGGAGAAGGTGTG	195
Ulk2	Forward: TATTGCAATGGTGGAGACCTGG Reverse: CTGCCGCAATCTGATGGAGA	94
Gapdh	Forward: GGTGGACCTCATGGCCTACA Reverse: CTCTCTTGATCAGTGTCCTTGCT	82
Rplp0	Froward: GGACCCGAGAAGACCTCCTT Reverse: GCACATCACTCAGAATTTCAATGG	85

qRT-PCR, quantitative reverse transcription polymerase chain reaction; Bp, number of base pairs; GAPDH, glyceraldehyde 3-phosphate dehydrogenase.

Gene expression was normalized to the geometric mean of *Rplp0* and *Gapdh* genes in line with Lin et al. (18). Relative expression levels were calculated using the 2^−ΔΔCT^ method. All samples were assayed in duplicate reactions.

### 2.4 Western blot analysis

Proteins were extracted from half uterine horns of 1-month-old mice only, using RIPA buffer supplemented with protease and phosphatase inhibitors (Roche). Western blot analyses were intentionally restricted to the early (1-month) time point to prioritize the initiation phase and because tissue availability for protein assays was insufficient at 3 months. Protein concentrations were determined with a protein assay kit (Bio-Rad Laboratories). Equal protein amounts were denatured and separated by SDS–polyacrylamide gel electrophoresis then transferred onto a polyvinylidene difluoride membrane (PerkinElmer). Membranes were blocked with 5% non-fat milk for 2 hours at room temperature and incubated overnight at 4 °C with primary antibodies (**Table 2**). After washing, HRP-conjugated secondary antibodies were applied for 1-hour at room temperature. Detection was performed using enhanced chemiluminescence (ECL kit, PerkinElmer) and imaged on a LAS4000 imager (Fujifilm). Protein band intensities were quantified using QuantityOne Analysis software and normalized to GAPDH or actin as a loading control. Data were presented as fold-change compared to the control group. For AKT, NFkB and MAPK1, the ratio of phosphorylated to total protein was calculated prior to fold-change determination.

**Table 2:** Antibody used for Western blot.

Target (Antigen)	Dilution	Company	# Catalog
LC3B	1/1000	Novusbio	nb100-2220
BCL2	1/1000	Cell signaling	3498
BCLXL	1/1000	Cell signaling	2764
P-AKT	1/1000	Cell signaling	9271
AKT	1/1000	Cell signaling	9272
BAX	1/1000	Cell signaling	14796
MAPK1	1/1000	Cell signaling	9102
P-MAPK1	1/1000	Cell signaling	9101
NFkB1	1/1000	Cell signaling	8242
P-NFkB	1/1000	Cell signaling	3031
Caspase3	1/1000	Cell signaling	9661
P62	1/1000	MBL	P045
GAPDH	1/10 000	Chemicon international	mab374
ACTIN	1/1000	SIgma	A2066

### 2.5 Immunohistochemistry and immunofluorescence

Serial uterine sections were immune-stained to (i) quantify LC3B as an autophagy-associated marker across uterine compartments and (ii) precisely localize glands relative to myometrial depth when H&E-based grading was equivocal, using αSMA/EpCAM double immunofluorescence.

Mouse and human uterine samples were deparaffinized, rehydrated, and subjected to antigen retrieval using an autoclave (11 min, 126 °C, 1.3 bar). After cooling down for 20 min, endogenous peroxidase activity was blocked by incubating the sections in 3% hydrogen peroxide for 20 min at room temperature (RT). Non-specific binding was blocked with Animal-Free Blocking Solution (Cell Signaling, 20 min at RT). Primary antibodies were diluted in REAL antibody diluent (Dako). Sections were incubated 1h at RT for anti-αSMA/FITC (Sigma, F377, 1/400) and anti-EpCAM (Cell Signaling, 93790, 1/400), or overnight at 4 °C with anti-LC3B (Cell signaling 3868, 1/1500). This antibody detects total LC3B and does not allow discrimination between LC3-I and LC3-II. After PBS washout, sections were incubated with the secondary antibody linked to horseradish peroxidase (ENVISION/HRP ready to use, Dako) for 30 min at RT.

For IHC, sections were incubated with Envision HRP-linked secondary antibody (Dako), revealed with DAB+ (Dako), counterstained with hematoxylin, and mounted with Entellan (Sigma-Aldrich). Slides were scanned using a NanoZoomer 2.0HT digital scanner (Hamamatsu). LC3B expression was identified as nucleo-cytoplasmic brown staining.

For IF, LC3B signal was amplified with fluorescein tyramide (PerkinElmer, 10 min), then mounted with DAPI Fluoromount-G (SouthernBiotech) and scanned with a SLIDEVIEW VS200 slide scanner (Olympus). For α-SMA/EpCAM double immunostaining, fluorescence amplification was performed similarly with tyramide, and slides were mounted and imaged under the same conditions.

### 2.6 Classification and grading of adenomyotic lesions

To evaluate the extent and severity of adenomyotic infiltration, lesions were graded according to the criteria of Bird et al. (19), based on the maximal depth of myometrial infiltration by ectopic endometrial glands and stroma on hematoxylin and eosin (H&E) stained sections. For each uterine horn, twelve serial sections were systematically examined at x20 magnification, and the section showing the deepest extent of adenomyotic foci was used to assign the grade. Grade 1: invasion limited to the inner one-third of the myometrium; grade 2: invasion extending into the inner two-thirds; grade 3: invasion through the full myometrial thickness. In cases where H&E grading was uncertain (e.g., distorted architecture, or subtle stromal invasion in 1 month-old mice), matched sections stained for α-SMA (smooth muscle) and EpCAM (epithelium) by immunofluorescence were reviewed to confirm gland position. This approach increased sensitivity while preserving comparability with the H&E-based system. Overall, this semi-quantitative grading method provided a reproducible measure of adenomyosis severity, serving as a baseline to compare lesion progression across experimental conditions.

### 2.7 Quantification of LC3B staining

Quantification of LC3B-IHC staining was performed using an H-score method. Staining intensity was classified into four categories (**Supplementary Figure 1**), the percentage of positively stained cells was estimated across multiple representative fields. The H-score was calculated as: (0 x % cells with intensity 0) + (1 x % cells with intensity 1) + (2 x % cells with intensity 2) + (3 x % cells with intensity 3), yielding a score ranged from 0 (no staining) to 300 (100% of cells with strong staining).

For immunofluorescence staining, image analysis and quantitative measurements were performed using MATLAB R2024b (MathWorks). Fluorescence images of transverse uterine sections were acquired in the RGB (red, green, blue) color space. In these images, LC3B, α-SMA, and DAPI signals were visualized in the red, green, and blue channels, respectively. Regions corresponding to lesions, eutopic endometrium, glandular and luminal epithelium, were manually delineated, and a binary mask was generated for each region. Stroma was deduced by substracting luminal and glandular epithelia from the eutopic endometrium. Subsequently, the RGB images were separated into their individual channels, and the red channel—containing the LC3B staining—was automatically segmented using MATLAB’s graythresh function. This procedure produced a binary image in which LC3B-positive areas appeared white (pixel value = 1) on a black background (pixel value = 0). Finally, the area (in mm²) corresponding to LC3B-positive staining within each mask was quantified.

All statistical analyses were performed using GraphPad Prism 10.1.1 (GraphPad Inc). Data normality was evaluated using the Shapiro-Wilk test and visually confirmed with QQ plot. Normally distributed data were analyzed with Welch’s t-test and are presented as mean ± SD, while non-normally distributed data were analyzed with the Mann-Whitney U-test and are reported as median with interquartile ranges (IQR). Statistical significance was set at P<0.05 and indicated as *P < 0.05, **P < 0.01, ***P < 0.001.

## 3 Results

### 3.1 Adenomyosis severity increases with age

**Figure 1:**
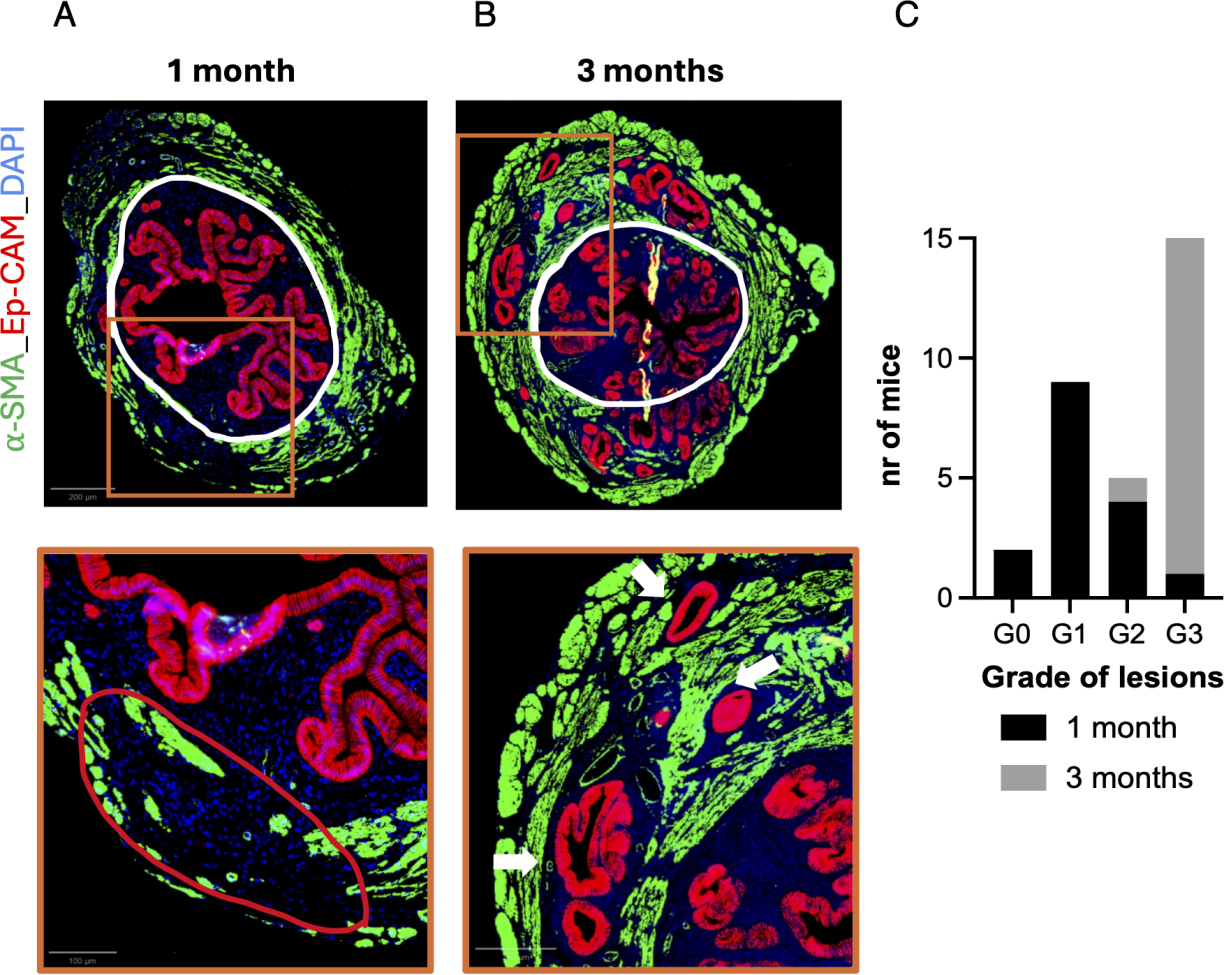
Severity of adenomyosis increases with age in mice. Histological and quantitative analysis of adenomyotic lesions in uteri from 1-month and 3-month-old mice. **(A)** Representative transverse uterine sections from adenomyosis mice at 1 month and **(B)** at 3 months with corresponding zooms (orange square) on area of interest. The white line delineates the border between eutopic endometrium and myometrium. Sections were stained by immunofluorescence with EpCAM (red, endometrial glands), α-SMA (green, smooth muscle cells), and DAPI (blue, nuclei). Adenomyosis foci (white arrows) are defined by the presence of glands and stroma within the myometrium. At 1 month, lesions were mild and focal, occasionally showing stromal invasion and partial distortion of myometrial organization (red circle). At 3 months, lesions were diffuse, multifocal, and deeply infiltrative. **(C)** Quantification of lesion severity based on depth of myometrial infiltration: grade I: inner third; grade II: inner two-thirds; grade III: entire thickness. Data are shown as absolute counts (n = 16 for 1 month; n = 15 for 3 months).

Histological analysis of uterine sections revealed a clear age-dependent differences in lesion severity (**Figure 1**) between 1-month and 3-month-old mice.

At 1 month, tamoxifen-treated mice (ADM, n = 16) showed early or mild disease. Thirteen percent of mice showed no detectable lesions (grade 0), 56% had grade 1 lesions, 25% grade 2 lesions, and only 6% exhibited grade 3 lesions. Even in mice without overt lesions, early disruption of myometrial architecture was observed, including loss of the normal inner circular and outer longitudinal layers. Occasional focal stromal invasion without glandular elements was also detected, consistent with the earliest stages of adenomyosis development (**Supplementary Figure 2**).

By 3 months, lesions were more severe and widespread. Nearly all tamoxifen-treated mice (ADM, n = 15) exhibited extensive grade 3 adenomyosis (93%), with the remaining animals showing grade 2 lesions (7%). These lesions were diffuse, multifocal, and infiltrated the full thickness of the myometrium.

### 3.2 Modulation of autophagy and apoptosis pathways in the early phase of adenomyosis

#### 3.2.1 Gene expressions are modulated at one month but not at 3 months

**Figure 2:**
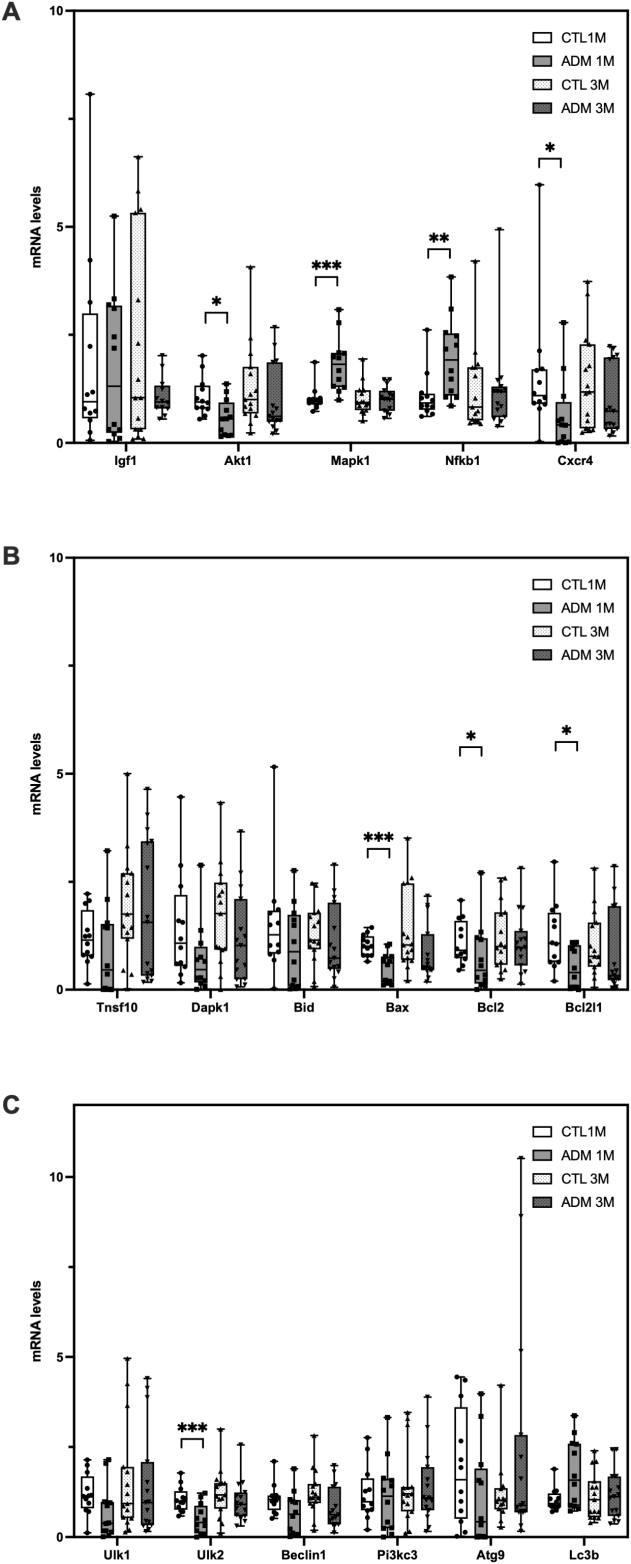
mRNA expression of autophagy-, apoptosis-, and signaling-related genes in adenomyosis. Relative mRNA expression levels of **(A)** signaling-, **(B)** apoptosis- and **(C)** autophagy- related genes in uterine tissues from control (CTL) and adenomyosis (ADM) mice at 1 and 3 months. Gene expression was normalized to *Gapdh* and calculated using the ΔΔCt method. Data are presented as median ± min-max. Statistical comparisons were performed using Welch’s *t*-tests after confirming normality, or Mann–Whitney tests otherwise. *p < 0.05; **p < 0,01; ***p < 0,001.

At 1 month, RT-qPCR revealed significant modulation of a subset of autophagy- and apoptosis-related genes, including *Akt1, Mapk1, Nfkb1, Cxcr4, Bax, Bcl2, Bcl2l1 and Ulk2* (**Figure 2**). No significant differences were observed for *Igf1, Tnfsf10, Dapk1, Bid*, *Ulk1, Beclin1, Pik3c3, Atg9 or Lc3b*.

By 3 months, expression levels of all tested genes were comparable between ADM and control groups, with only a non-significant trend toward lower expression in ADM mice (**Figure 2**).

#### 3.2.2 Protein expression of LC3B, MAPK and BAX are modulated at 1 month

**Figure 3:**
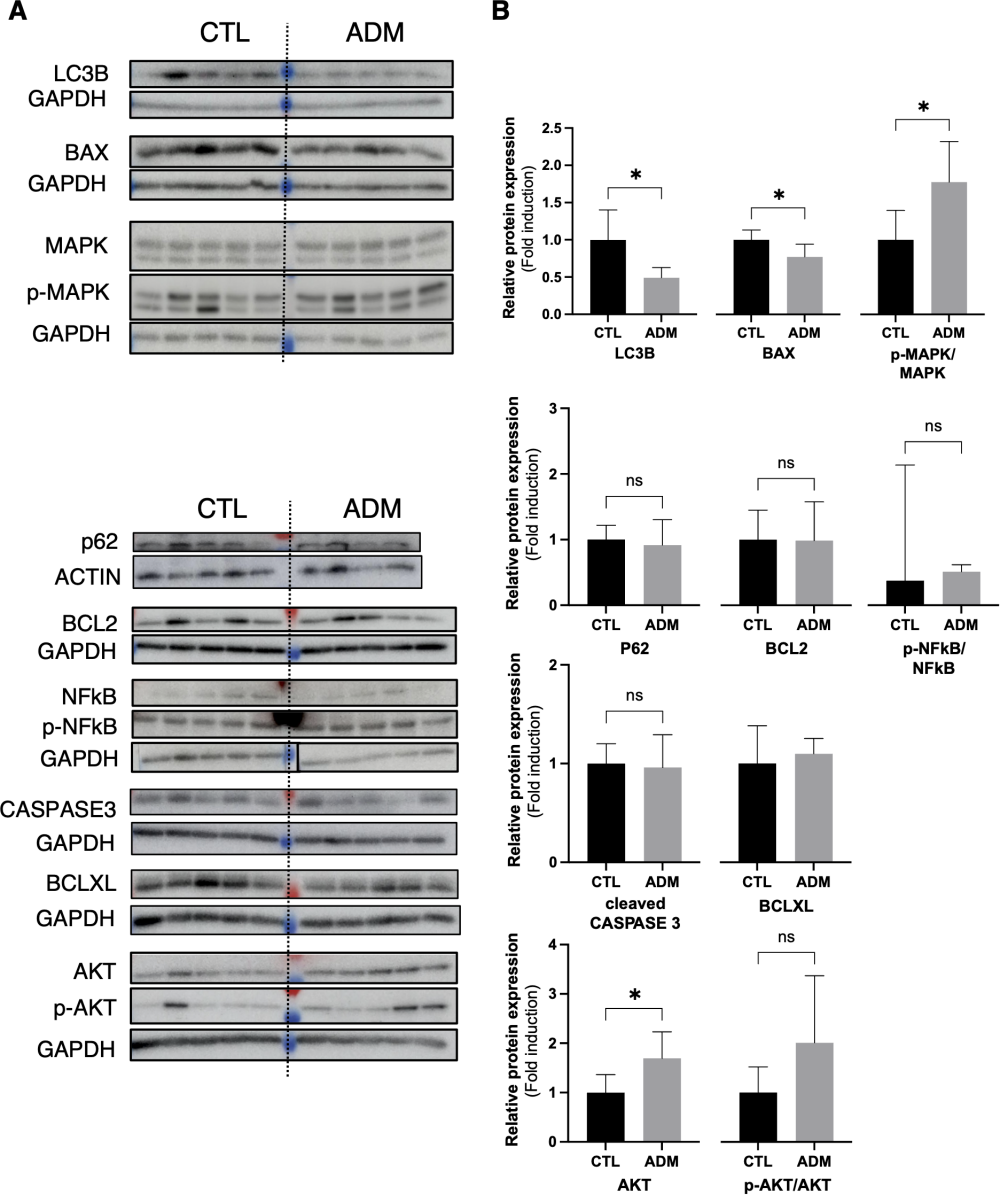
Altered expression of autophagy- and apoptosis-related proteins in early adenomyosis. **(A)** Representative Western blots of LC3B, BAX, total and phosphorylated-MAPK (p-MAPK), P62, BCL2, total and phosphorylated NF-κB (p-NF-κB), cleaved caspase-3, BCL-XL and total and phosphorylated AKT (p-AKT) in uterine tissues from control (CTL) and adenomyosis (ADM) mice at 1 month (n = 5 per group), except for p62 (ADM group: n = 4 due to insufficient protein quantity for Western blot analysis). GAPDH or actin served as a loading control. **(B)** Quantification of relative protein levels normalized to GAPDH or actin. For MAPK, NF-κB and AKT, kinase activation was evaluated using phosphorylated-to-total protein ratios prior to fold-change determination. Data are presented as mean ± SD for normally distributed variables (LC3B, BAX, MAPK, P62, BCL2, cleaved caspase 3, BCL-XL and AKT) and as median ± IQR for non-normal variables (NF-κB). Group comparisons were performed using Welch’s t-tests or Mann–Whitney tests as appropriate. *p < 0.05; ns: not significant. Changes in total protein abundance were interpreted cautiously, as they may reflect altered protein turnover rather than kinase activation.

Western Blot analysis at 1 month confirmed significant modulation of p-MAPK/MAPK and BAX protein levels in adenomyosis mice compared with controls. In addition, LC3B protein levels were significantly altered despite the absence of transcriptional changes at the mRNA level (**Figure 3**). Total AKT protein levels were normalized to GAPDH, and kinase activation was assessed using phosphorylated-to-total ratios; however, no significant differences were observed for p-AKT/AKT. Although total AKT levels appeared increased in ADM mice, these changes were interpreted cautiously, as they may reflect altered protein turnover rather than changes in kinase activation. No significant differences were observed for p62, BCL-2, BCL-XL, p-NFkB/NF-KB, or cleaved caspase-3 (**Figure 3**).

Given the absence of transcriptional modulation at 3 months, protein analyses were limited to 1-month samples.

#### 3.2.3 Reduced LC3B staining in the uterus of younger mice

**Figure 4:**
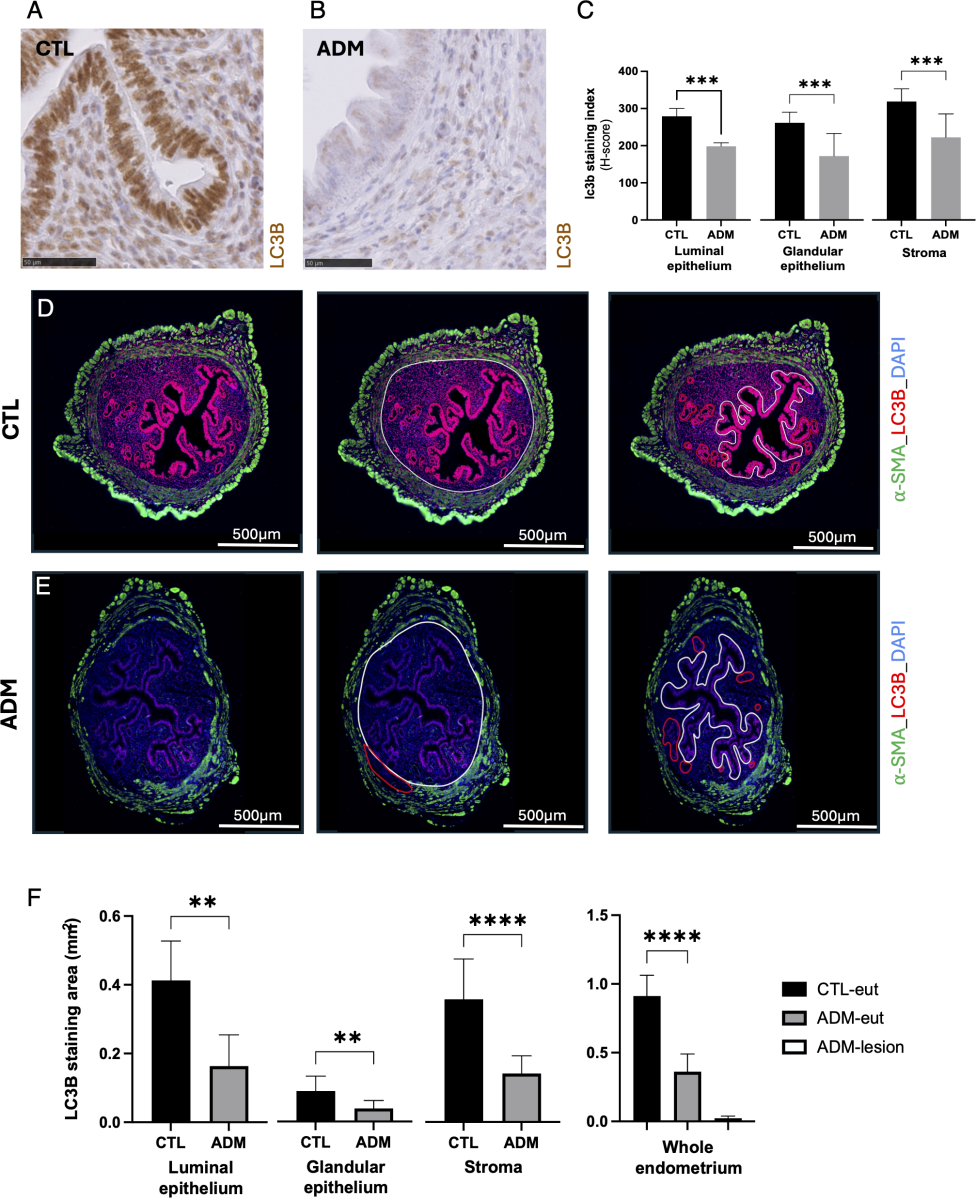
Decreased LC3B expression in the uteri of adenomyosis mice. **(A, B)** Representative images of LC3B expression by immunohistochemistry in uterine transverse sections from 1 month control **(A)** and adenomyosis **(B)** mice. **(C)** Quantification of lc3b staining index (H-score) across luminal epithelium, glandular epithelium, and stroma. **(D, E)** Representative images of LC3B expression by immunofluorescence (LC3B red; α-SMA green; DAPI blue) in uterine sections from control **(D)** and adenomyosis **(E)** mice. From left to right: original transverse uterine section; delimitation of eutopic endometrium (white line) and lesions (red lines); delimitation of luminal (white line) and glandular epithelium (red lines) within the eutopic endometrium. Stroma was identified by subtracting luminal et glandular epithelium from total eutopic endometrium. **(F)** Quantification of LC3B stained area in different compartments of eutopic endometrium: luminal epithelium, glandular epithelium, and stroma and across eutopic and ectopic endometrium. Data are presented as mean ± SD. Group comparisons were performed using Welch’s t-test after confirming normality. **p < 0.01; ***p < 0.001; ****p < 0.0001; ns: not significant. N = 12 in each group.

Immunohistochemistry at 1-month showed a significant reduction of LC3B staining in the endometrium of adenomyosis mice compared with controls (**Figures 4A-C**). In parallel, immunofluorescence quantification revealed a similar trend with an even more pronounced decrease of LC3B-positive area in the stromal compartment (**Figures 4D-F**).

**Figure 5:**
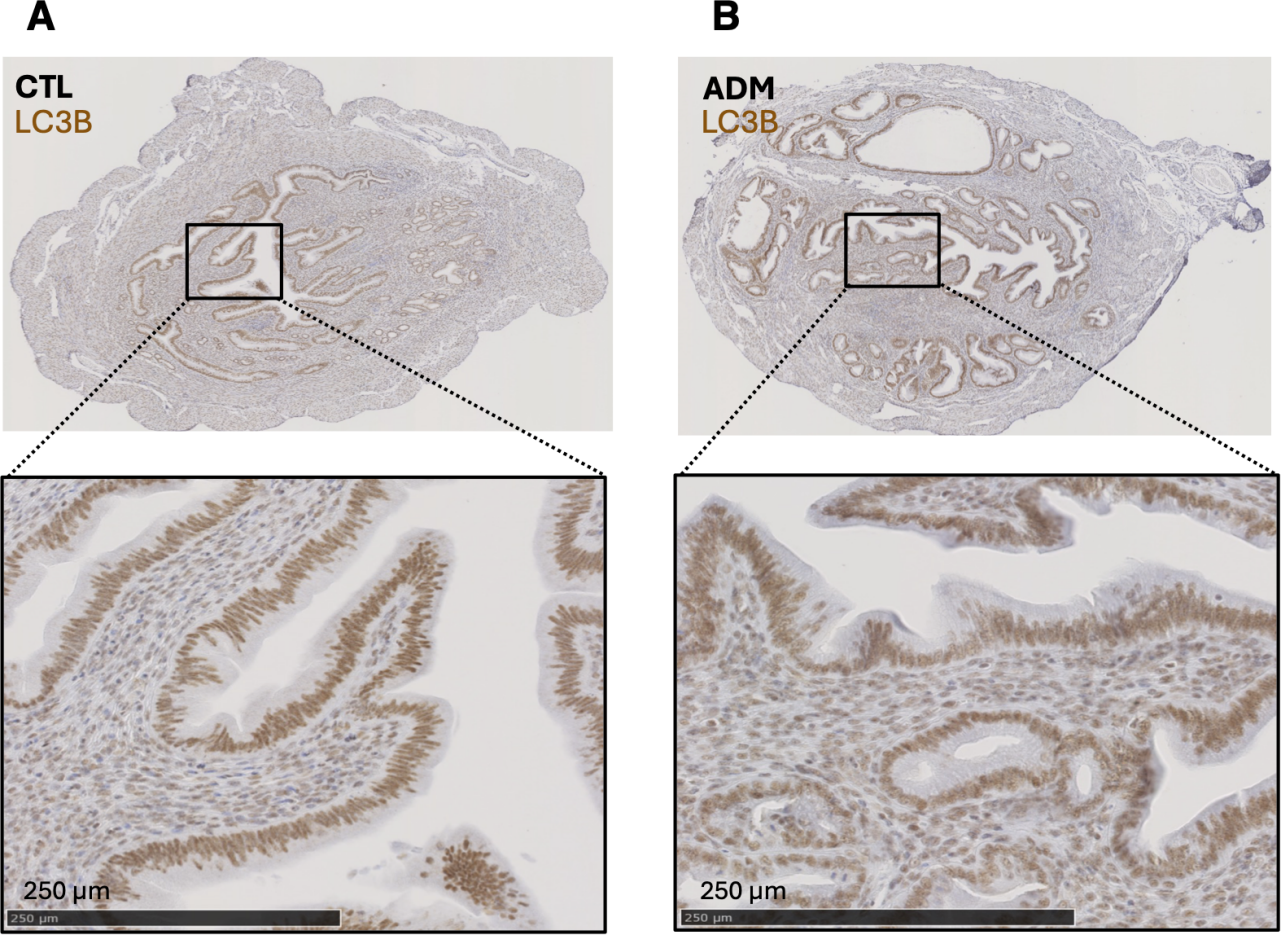
LC3B expression in 3-month-old adenomyosis mice. Representative IHC images of LC3B expression in uterine sections from control **(A)** and adenomyosis **(B)** mice. For each image, focus on a zone including luminal epithelium and stroma. No significant difference in lc3b staining intensity was observed between groups.

At 3-months, LC3B staining was comparable between adenomyosis and control groups by the assay used (**Figure 5**).

### 3.3 Decreased LC3B staining in the stroma of human adenomyosis lesions

**Figure 6:**
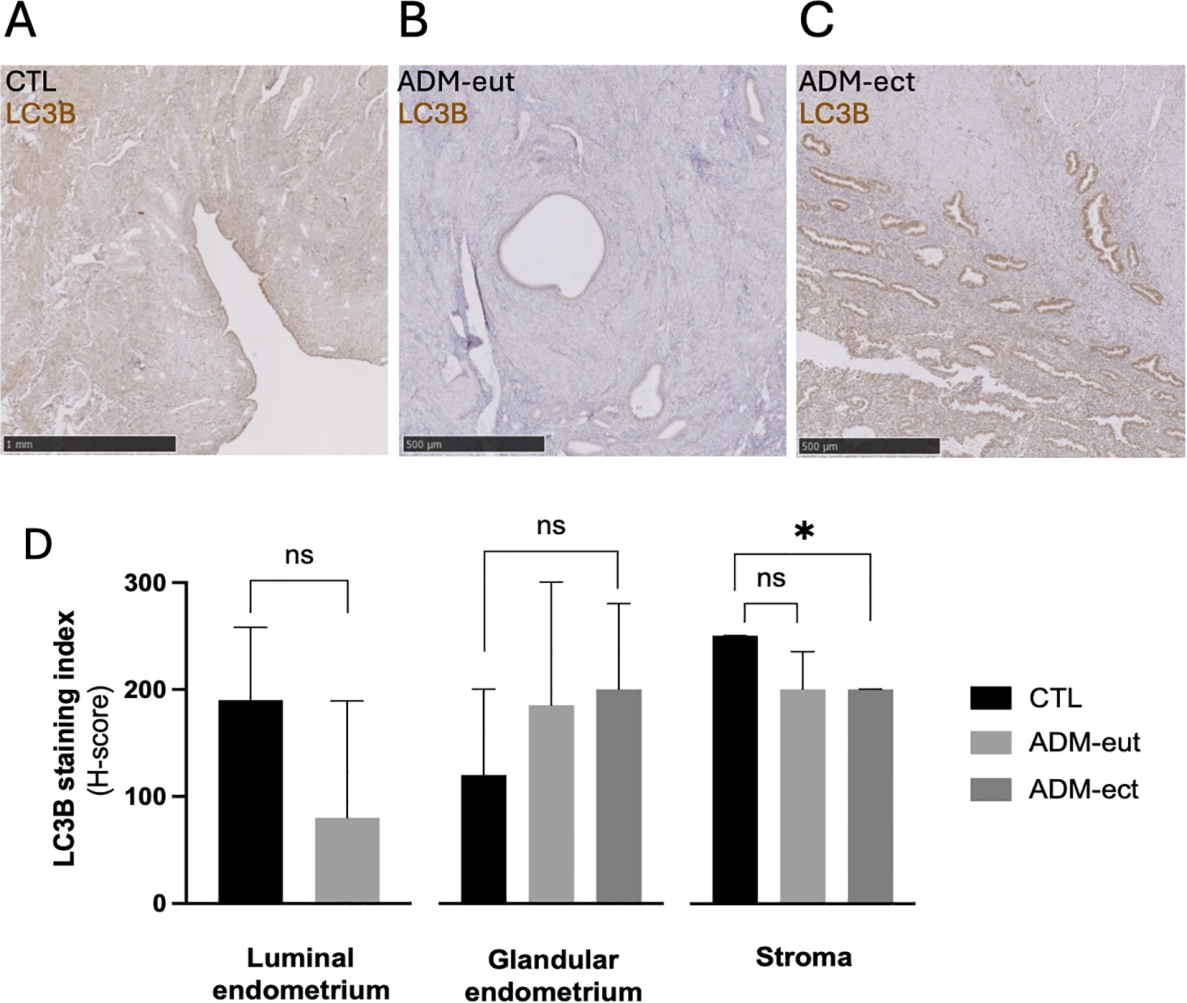
Decreased stromal LC3B expression in human adenomyosis lesions. Representative IHC images of LC3B expression in human endometrial tissue from control women **(A)**, eutopic endometrium of adenomyosis patients **(B)**, and ectopic endometrial lesions **(C)**. **(D)** Quantification of LC3B staining index (H-score) in luminal epithelium, glandular epithelium, and stroma. Data are presented as mean ± SD for normally distributed variables (luminal epithelium) and as median ± IQR for non-normal variables (glandular epithelium, stroma). Group comparisons were performed using Welch’s t-tests or Mann–Whitney tests as appropriate. *p < 0.05; ns: not significant.

LC3B expression was evaluated in human uterine samples (n = 12 adenomyosis, n = 10 controls; **Figure 6**). Endometrium from control patients showed robust LC3B staining across luminal epithelium, glandular epithelium, and stroma. In adenomyosis specimens, ectopic lesions displayed a selective and significant reduction of LC3B staining in the stromal compartment, while glandular epithelium retained expression levels comparable to controls. The eutopic endometrium of adenomyosis patients also showed LC3B expression levels comparable to controls.

## 4 Discussion

This study identifies autophagy-associated alterations markers as a temporally and spatially regulated process in adenomyosis, based on complementary mouse and human analyses. In the neonatal tamoxifen-induced CD1 model, autophagy- and apoptosis-related markers and pathways were significantly altered during the early phase of lesion establishment but were no longer detectable by the assays used at later stages. In contrast, human adenomyosis specimens displayed a persistent and selective reduction of LC3B staining in the stromal compartment, while epithelial components remained comparable to controls. Together, these findings suggest that early adenomyosis development in mice is associated with transient modulation of autophagy-associated markers, while in established human lesions, LC3B reduction is compartmentalized within stromal cells.

### 4.1 Clinical heterogeneity and alignment of our model with diffuse disease

Clinically, adenomyosis presents as a heterogeneous condition ranging from junctional-zone thickening to full-thickness infiltration, with focal or diffuse phenotypes (20). Focal adenomyosis- including cystic adenomyoma- is often associated with deep endometriosis (21, 22), whereas diffuse adenomyosis consists of multifocal gland-stroma aggregates throughout the myometrium (19). Our tamoxifen-induced CD1 model reproduces this diffuse pattern: lesions arise at the endometrial–myometrial interface (EMI) and extend centripetally into the myometrium, consistent with the basalis-invagination hypothesis and recent NGS-based clonal evidence (5).

Several pathogenetic frameworks have been proposed, including invagination of basalis endometrium across a disrupted junctional zone, metaplasia of Müllerian remnants or progenitor cells and endometrial-myometrial disruption (EMID) after iatrogenic injury (23, 24). The EMID hypothesis is supported by experimental mouse models and by epidemiological associations with uterine procedures such as curettage or cesarean delivery (25). Although our model induces adenomyosis pharmacologically rather than mechanically, both approaches converge on interface instability, a context in which the early autophagy dampening observed here could promote stromal survival and invasion (7).

Viewed together, this clinical heterogeneity has a practical consequence: phenotype matters. The autophagy and signaling features described in our study are aligned with diffuse adenomyosis and may not generalize to focal phenotypes. In particular, focal adenomyosis located in the outer myometrium (FOAM) could involve distinct migratory routes, hormonal milieus, or mechanical constraints (21, 26). This phenotypic diversity plausibly underlies conflicting reports in literature. Future investigations should therefore stratify by subtype, incorporate compartment-resolved endpoints (stromal *vs* epithelial; eutopic *vs* ectopic), and align sampling with cycle phase and hormonal exposure when interrogating autophagy and related pathways.

### 4.2 Early modulation of autophagy-associated markers during adenomyosis initiation

Our findings indicate that early stages of adenomyosis development are associated with a modulation of autophagy-associated markers, coinciding with myometrial disorganization and initial stromal invasion. In tamoxifen-exposed uteri, smooth-muscle layers were disrupted, creating permissive planes for endometrial invasion. However, C57BL/6J mice can exhibit similar remodeling without developing adenomyosis, suggesting that additional molecular cues are required (25). Acquisition of invasive properties, through epithelial-mesenchymal transition (EMT), a process promoted by hyperestrogenic conditions, has been implicated in both human and preclinical models (4, 27). Estrogen is known to restrain autophagic activity, which may contribute to the early reduction of LC3B observed in our model, without allowing conclusions on autophagic flux (11). We used cycle synchronization via estrogen administration, which could have affected the autophagic activity monitored in our research. However, both adenomyosis and control group received identical hormonal treatment, mitigating potential bias. During estrus, autophagy is dampened but not completely inhibited, which is why autophagic variation can still be monitored during this phase.

Beclin1, a major autophagy initiator, connects autophagy and apoptosis through its interaction with Bcl-2 (28). Reduced Beclin-1 expression is associated with proliferation, migration, and EMT via PI3K/AKT/mTOR activation, while its overexpression enhances apoptosis (13, 27). Consistent with this, we observed early modulation of apoptotic regulators such as *Cxcr4, Bax* and *Mapk*, supporting the existence of an early stress-responsive state in which autophagy-associated components, apoptosis-related pathways and signaling networks intersect during lesion initiation. The absence of cleaved caspase-3 modulation suggests that apoptotic execution is not fully engaged at this stage, potentially allowing survival of invasive stromal cells without indicating a shift toward cell death. In other systems, autophagy and apoptosis are bidirectionally coupled, and dysregulated autophagy can trigger caspase-independent cell death through ERK/MAPK signaling (29–31).

Interestingly, *Lc3b* mRNA was unchanged at 1 month, whereas Western blot and immunohistochemistry consistently showed reduced LC3B protein, indicating a dissociation between transcriptional and protein-level regulation of this autophagy-associated marker (32–34). In addition, total p62 protein levels were not significantly different between control and adenomyosis groups. Because p62 is degraded during autophagic flux and its accumulation is commonly associated with impaired autophagic degradation (35, 36), the absence of p62 accumulation does not support the presence of a classical lysosomal blockade in our model. Taken together, these findings suggest that early adenomyosis development is associated with modulation of autophagy-associated markers rather than clear evidence of impaired of autophagic degradation. This divergence underscores the complexity of autophagy regulation beyond transcriptional control and highlights the limitation of static markers such as LC3B in inferring autophagic activity (35). Post-transcriptional mechanisms or altered protein turnover may explain reduced LC3B protein levels despite stable transcripts. Similar mRNA-protein discrepancies have been reported in endometriosis and cancers (37, 38), emphasizing the need for dynamic autophagy assays to determine functional autophagic activity, which were beyond the scope of the present study (39).

In addition, LC3B immunostaining was observed in both cytoplasmic and nuclear compartments (**Figure 4**). Although LC3B is classically considered a cytoplasmic marker of autophagosomes, a distinct nuclear pool of LC3 has been described (40, 41). In the absence of subcellular fractionation or dynamic assays, the present data do not allow discrimination between altered autophagosome formation, subcellular redistribution, or flux modulation. The nuclear staining observed should therefore be interpreted cautiously within the broader context of autophagy-associated marker modulation.

### 4.3 Persistent stromal-specific reduction of LC3B in human adenomyosis

Interestingly, in one month old mice, LC3B staining area is strongly dampened in the stromal compartment. However, by three months in the murine model, no transcriptional or LC3B staining differences were detectable between adenomyosis and control uteri by the assays used. This may reflect biological adaptation, engagement of alternative pathways such as angiogenesis, fibrosis, or chronic inflammation or dilution of compartment-specific effects in whole-tissue analyses. Collectively, these data indicate that early murine adenomyosis development is associated with transient modulation of autophagy-associated markers, without evidence of sustained autophagic impairment based on the assays performed.

In contrast, human adenomyosis samples displayed a persistent stromal-specific reduction of LC3B, whereas glandular epithelium and the eutopic endometrium showed levels comparable to controls. This observation, consistent with reports of compartment-specific autophagy defects in endometriosis (42–44), highlights stromal cells as a critical niche for disease persistence, given their roles in matrix remodeling, cytokine signaling, and invasive behavior (45–47). Sustained downregulation of autophagy-associated markers may thus contribute to stromal survival, apoptosis resistance, and tissue remodeling in established lesions.

Mechanistic links have been proposed. Decreased expression of TSC2, LC3B and mTOR expression has been reported in human endometrium, activating mTORC1, suppressing autophagy, and enhancing proliferation and EMT (48). Beclin-1 is also reduced in eutopic endometrium from adenomyosis patients and correlates inversely with CA125 levels and pain scores (49). Together, these findings reinforce the plausibility of autophagy-related alterations as contributors to lesion persistence.

However, the literature remains heterogeneous: while many studies report reduced LC3B or Beclin1 in adenomyosis (43, 44, 48, 49), others describe no change (50) or even increased autophagy markers expression (51–54). Such variability likely reflects differences in disease stage (early *vs* established disease), compartment analyzed (stromal *vs* epithelial *vs* whole tissue), menstrual phase, phenotype, or technical approaches. Future work should therefore stratify by phenotype and cycle stage and integrate flux-based assays to resolve whether stromal autophagy impairment consistently underlies disease maintenance.

### 4.4 Limitations & perspectives

This study has several limitations that should be considered when interpreting the findings. First, gene and protein analyses were performed on whole uterine tissue, which may dilute cell-type restricted effects. Compartment-specific alterations might therefore be underestimated and static whole-tissue measurements may not fully capture spatially restricted autophagy-associated regulation. Future work should incorporate laser-capture microdissection, stromal/epithelial cell sorting, or single-cell/spatial transcriptomic and proteomic approaches.

Second, autophagy in the endometrium is tightly regulated by cycle phase. In this study, mice were synchronized with estradiol and analyzed specifically in proliferative states to reduce inter-animal variability. While this approach increases internal consistency, it may also mask phase-dependent fluctuations in autophagy. Future work should therefore adopt phase-stratified designs in both mice (e.g., estrus *vs* diestrus) and humans (proliferative *vs* secretory endometrium) to fully capture hormonal influences on autophagy regulation.

Third, conclusions about autophagy relied on static measurements (LC3 and p62) rather than assessment of autophagic flux. Although p62 protein expression was evaluated, static levels alone are insufficient to determine dynamic flux activity (38). Definitive validation would require *in vivo* flux assays, such as p62/LC3 turnover under lysosomal inhibition or the use of fluorescent LC3 reporter mice. Complementary ex vivo approaches using endometrial organoids or primary stromal cell cultures could provide additional mechanistic insight under controlled hormonal conditions (55).

Fourth, the tamoxifen-induced CD1 mouse reproduces diffuse adenomyosis but does not fully capture the chronicity, heterogeneity, or menstruation-linked physiology of human disease, which limits generalizability. Validation in complementary systems including human endometrial organoids, would strengthen translational relevance.

Fifth, the human cohort was limited in size and confounded by age differences and by the context of gender-affirming surgery for controls, with potential exposure to exogenous hormones. Larger cohorts with age-matched controls and detailed hormonal information are warranted to strengthen these findings.

Finally, Western blot analysis was not extended to 3-month samples since gene expression levels were already normalized at this stage. However, transcriptional stability does not preclude potential post-transcriptional or post-translational regulation, and the absence of detectable differences at this time point reflects the limits of the assays performed rather than definitive evidence of restored autophagic regulation.

Despite these limitations, our study has notable strengths. It combines a well-characterized experimental model with human validation, applies complementary readouts at transcript, protein, and histological levels, and integrates a temporal dimension by analyzing pre-lesional (1 month) and lesion-established (3 months) stages. This design enabled us to distinguish early, transient changes in mice from persistent, compartment-specific alterations in human lesions.

Together, these strengths and limitations outline a clear path forward: future studies should apply cell type-resolved analyses across defined hormonal contexts, integrate autophagic flux assays *in vivo* and ex vivo, and validate findings in larger human cohorts. Such approaches will clarify whether stromal autophagy-associated dysregulation consistently contributes to adenomyosis persistence and could be exploited as a therapeutic target.

## 5 Conclusion

Across complementary mouse and human analyses, this study identifies autophagy-associated alterations as a temporally and spatially constrained feature of adenomyosis. In the tamoxifen-induced CD1 model, modulation of autophagy-associated markers was early and transient, coinciding with myometrial disorganization and initial stromal invasion, but was no longer detectable by the assays used by three months. In contrast, human adenomyosis specimens showed a persistent stromal-specific reduction of LC3B, implicating the stroma as a potential key compartment for lesion maintenance.

The combined use of a time-resolved murine model and human validation represents a major strength, allowing us to distinguish early associations with lesion initiation from compartment-specific alterations in established disease. These findings highlight autophagy-associated regulation as a pathway of interest in adenomyosis pathophysiology and suggest that stromal autophagy-associated dysregulation may represent a targetable vulnerability.

Future work should integrate dynamic flux assays, cell type–resolved analyses, and larger, stratified human cohorts to determine whether altered autophagy regulation contributes to of disease persistence and represents a viable therapeutic entry point.

## Data Availability

The original contributions presented in the study are included in the article/**Supplementary Material**. Further inquiries can be directed to the corresponding author.
