# Supplementary figures and images for: Early modulation of autophagy-associated markers in murine adenomyosis and selective persistence in human stromal lesions
Source: Front Endocrinol (Lausanne). 2026 Mar 25;17:1750026. doi: 10.3389/fendo.2026.1750026 (PMC13056826; doi:10.3389/fendo.2026.1750026)

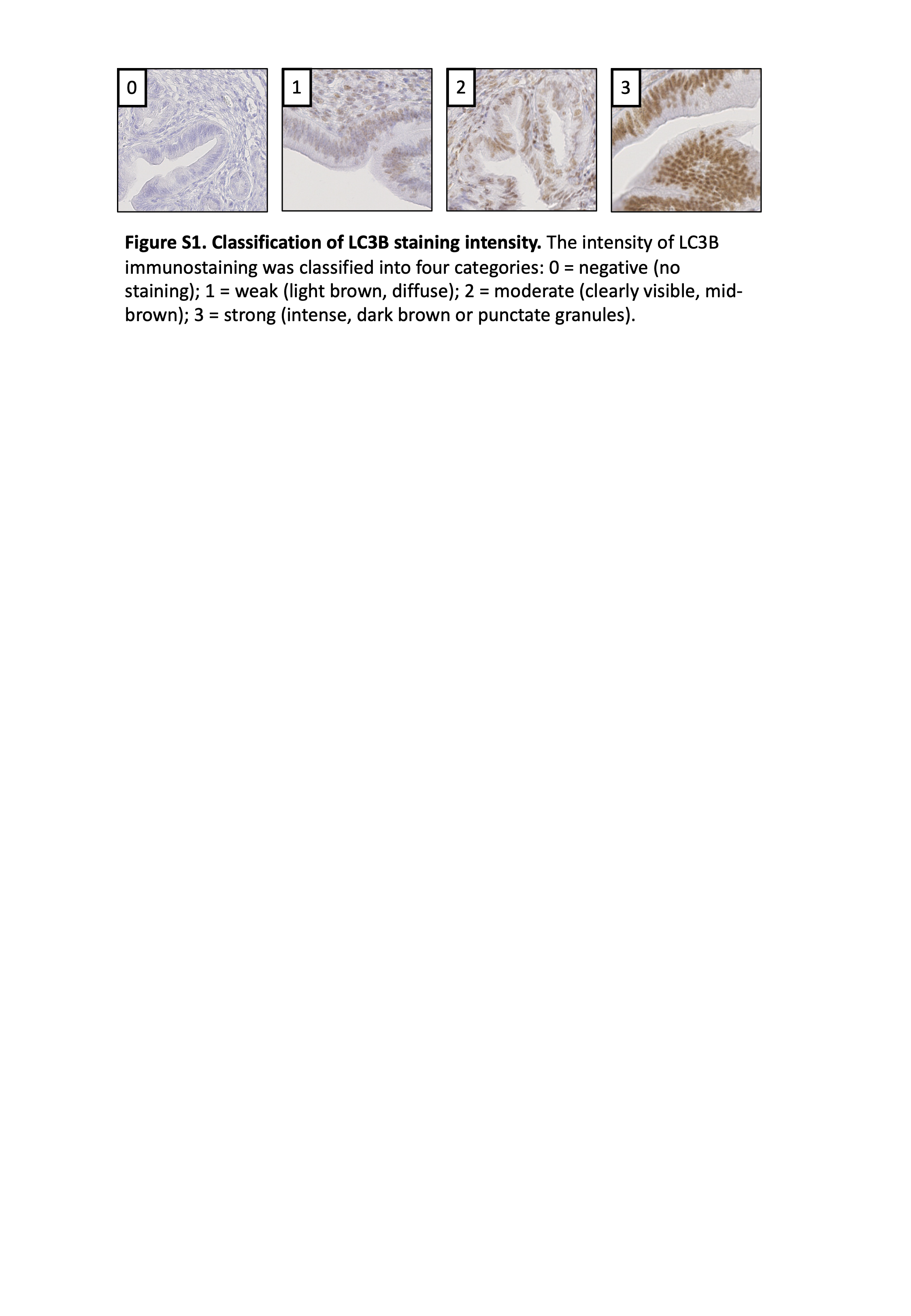

Supplement: Supplementary file 1 [file Image1.tiff]

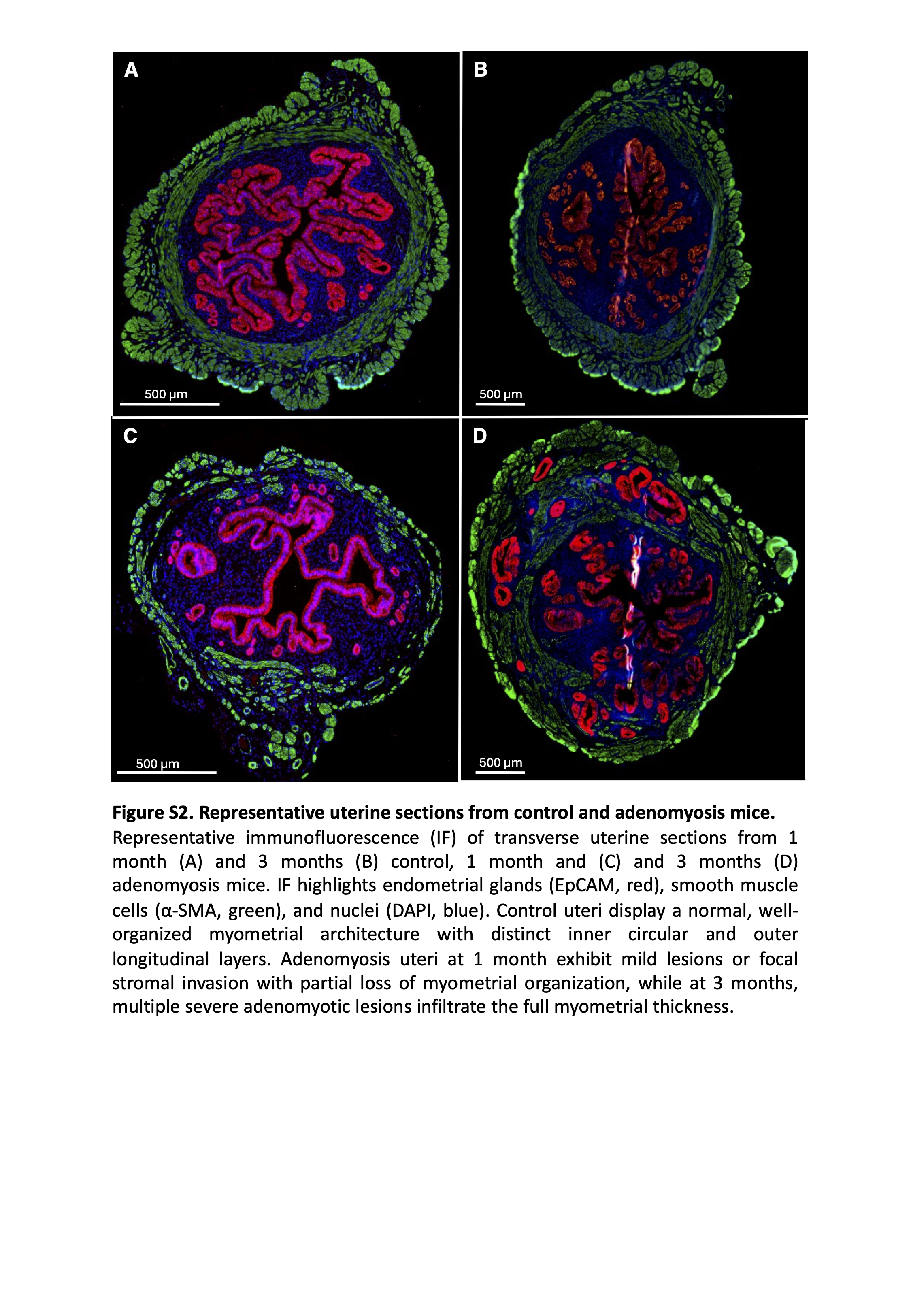

Supplement: Supplementary file 2 [file Image2.tiff]
